# Determination of mode strengths in channel waveguide from the complex electric field

**DOI:** 10.1038/s41598-024-80054-5

**Published:** 2024-11-19

**Authors:** Isaac Doughan, Atri Halder, Igor Reduto, Matias Koivurova, Timo Aalto, Matthieu Roussey, Jari Turunen

**Affiliations:** 1 Center for Photonics Sciences, University of Eastern Finland, P.O. Box 111, 80101 Joensuu, Finland; 2 VTT Technical Research Ctr. of Finland Ltd., Espoo, Finland

**Keywords:** Integrated optics, Optical techniques

## Abstract

We show that the mode strengths of a guided field in an arbitrary asymmetric channel waveguide can be uniquely determined from self-referencing interferometric measurements at the exit plane of the waveguide. This requires knowledge of both the amplitude and phase of the complex electric field distribution. Although the amplitude can be obtained from the measured intensity profile easily, the phase retrieval is usually non-trivial. We develop an innovative, alternative and promising technique, where the complex cross-spectral density (CSD) function is measured using a customized wavefront folding interferometer. We then construct the total electric field (complex valued), from which we can determine the strengths of the allowed modes for an asymmetric strip waveguide. Our retrieval algorithm also provides the phase information (intermodal dispersion) associated with each mode, directly from the measured electric field distribution. Moreover, we experimentally demonstrate the developed scheme for different in-coupling (butt-coupling) conditions, resulting in different modal strength distributions.

## Introduction

Applications of integrated photonics in fields such as sensing using fluorescence^1,2^, up- or down-frequency light conversion^3,4^, photonic computing^5^, and other low-photon sources^6^ require a precise understanding of photonic circuitry. Specifically, bus waveguides^7^ are used as a supporting platform for the functional building blocks, creating the necessary operations along the light path.

While designing a single-mode channel waveguide for a specific wavelength range is relatively easy, it becomes more challenging for very broadband operations, especially when light is generated inside the waveguide for source integration^8,9^. In such cases, several modes may establish, perturbing the signals and modifying the operation of different integrated elements along the circuit. Moreover, since only quasi-polarized modes, and not fully polarized modes, exist in a channel waveguide, signals at the same wavelength can interfere, yielding varied output responses. As a consequence, it is crucial to determine the strength of each mode in such a waveguide. In this article, we propose and present a straightforward yet unique approach to solving this problem.

Modes are ubiquitous across different branches of science. They can be found in many systems described by a partial differential equation supplemented with boundary conditions. A prime example of modal descriptions is usual continuous wave lasers based on stable resonators. Lasers can support multiple transverse modes, depending on the geometry of the laser cavity. Laser modes are said to be uncorrelated because they oscillate at different frequencies^10,11^, which leads to reduced spatial coherence. An algorithm to find the modal strengths of partially coherent lasers was introduced in Ref. ^12^ and experimentally verified in Ref. ^13^. An alternative way to determine the modal profiles and their relative strengths was presented in Ref. ^14^, where an edge-emitting semiconductor diode was chosen for the illustration.

However, modes excited in waveguides by spatially coherent light are correlated, and yield spatially coherent output light. Fortunately, there is also a way to determine the weights of mutually correlated modes. Knowledge of the complex electric field at the exit (or image) plane of the waveguide alone is sufficient to determine the weights of the modes. Mode strength determination of a multi-mode fiber, based on diffractive optical elements ^15^, SLM based digital holographic techniques ^16,17^ have been proposed and demonstrated. These techniques are based on overlap integral methods. In this article, we present an experimental procedure based on the measurement of the total electric field at the exit plane of a channel waveguide, enabling the determination of the strength of each mode of a multimode waveguide once coupled to a proper retrieval algorithm.

**Fig. 1:**
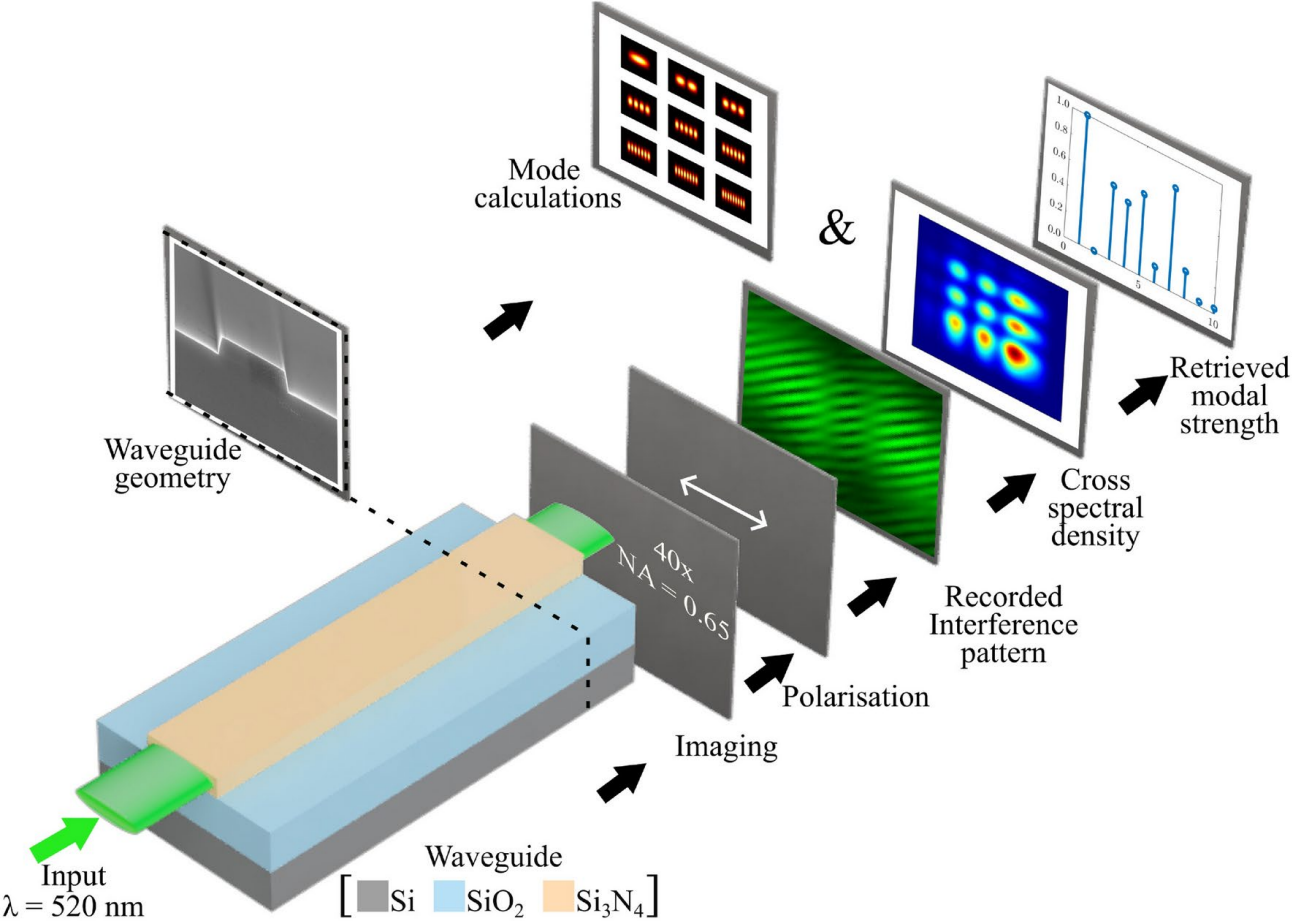
Conceptual overview of the mode strength retrieval process. A channel waveguide is fed with a 520 nm wavelength light source. The emerging light intensity is captured using a microscope objective, after which the transverse electric (TE) polarization is isolated and the interference pattern is recorded by a wavefront folding interferometer (WFI) for detailed wavefront analysis. From this analysis, the cross-spectral density is determined. The possible modes that the waveguide can support are calculated based on its geometric attributes and refractive index profile. By correlating these theoretically calculated modes with the acquired cross-spectral density, the modal strength of each mode is retrieved.

Several attempts have been made on determining the modal strengths of multimode/few-mode waveguides or fibers ^18–24^. In most cases, the modal strengths/weights are estimated from the total intensity at the output plane of the waveguide, while excited with different modal strength distribution. Although these approaches provide a fair estimate of the modal strengths, the solutions are not unique. Only complete knowledge of the total (complex-valued) electric field profile at the output can provide a unique solution.

This article deals with the direct retrieval of the strength of each individual mode sustained by a channel waveguide from the total electric field at the exit plane. In section “Concept” we present a conceptual overview of mode strength retrieval process. Section “Modal representations in waveguides” discusses modal representation in waveguides, explaining how modes are represented within the waveguide structure. In Section Complex field and modal strength retrieval, we delve into complex field and modal strength retrieval, presenting equations involving cross-spectral density as the basis of mode strength extraction, accompanied by simulation results. Finally, in Section Experimental results, we demonstrate the technique by presenting and discussing promising experimental results of the retrieved mode strengths.

## Concept

A conceptual illustration of the method to retrieve the strength of discrete modes at the output of a channel waveguide is presented in Fig. 1. We consider, as an example, a silicon nitride waveguide, especially designed to sustain several modes in the quasi-TE regime, and only the fundamental quasi-TM mode TM$_{00}$. The height of the waveguide is 330 nm, its width is 3 $\upmu$m, and the vacuum wavelength of the light coupled into the waveguide is 520 nm. We inject the light into the waveguide using end butt-coupling method. Immediately after the output, a linear polarizer separates TE from TM contribution. Only the intensity from the superposition of the TE modes is considered. The individual modes are spatially completely coherent, mutually orthogonal, as well as mutually correlated. One gets mutually uncorrelated modes when the individual modes have different frequencies, which is not the case here.

The total light enters a wavefront folding interferometer (WFI), detailed in Refs. ^25,26^, which measures the CSD by analyzing the interference pattern. The WFI creates two identical replicas of the same incident beam like field first. Next, in the arm-1 (arm-2) the beam wavefront folds along *x* (*y*) axis. Finally, from the superposition of the two folded beam like fields and by scanning those along the transverse direction one gets the CSD. The main difference between traditional WFI and our customized WFI is the design. However, we guide the readers to see Refs. ^26^ for the detailed description and working principle of the WFI, that we have used here. After decoding the output of the WFI using an appropriate algorithm, the weight of each mode can be retrieved. The algorithm uses the field distribution simulated for the studied waveguide, i.e., the field distribution of each mode is known a priori. The retrieval provides both the modal strengths as well as the inter-modal dispersion parameters.

## Modal representations in waveguides

In the following, we consider the strong hypothesis of a slab waveguide and scalar fields to describe the methodology. The following arguments can be extended to other waveguide designs and vectorial light as well. This is allowed because a polarizer is used at the output of the channel waveguide and because the result concerns only the output plane and not what happens inside the channel. In a waveguide with two-dimensional cross-section, the high and inherent birefringence could completely modify the modal strength distribution at different locations along the propagation.

Due to the orthogonality of modes in a slab waveguide, the amplitude and phase of the guided field $E_\textrm{out}$ at the exit (or image) plane of the waveguide may in general be represented as a superposition of the known modal fields $\phi _p(x)$, as in

1 $$\begin{aligned} E_\textrm{out}=\sum _{p=0}^{P-1} \alpha _p\phi _p(x), \end{aligned}$$

where we have left the frequency dependence implicit for brevity, *P* is the number of modes, and $\alpha _p$ are the generally complex valued modal weights with $\psi _p= \arg [\alpha _p]$. Since the waveguide modes are mutually orthogonal, they obey the relation

2 $$\begin{aligned} \int _{-\infty }^\infty \phi _p^*(x)\phi _q(x) \textrm{d}x = \delta _{pq}, \end{aligned}$$

where $\delta _{pq}$ is the Kronecker delta. Additionally, the modes $\phi _p(x)$ in slab waveguides are real-valued, although their superposition generally is not. Hence, by multiplying both sides of (1) by $\phi ^{*}_q(x)$, integrating over *x*, and using (2), we attain the weights from

3 $$\begin{aligned} \alpha _p = \int _{-\infty }^\infty E_\textrm{out}\phi _p(x)\textrm{d}x. \end{aligned}$$

This gives $\alpha _p\ne 0$ only for modes that propagate in the waveguide. There is also a continuum of radiation modes, but these are not of interest here. Note that (3) gives weights of the amplitudes of the modes, which are complex-valued numbers. The intensity weights

4 $$\begin{aligned} c_p = \left| \alpha _p\right| ^2 \end{aligned}$$

are of course real, and correspond to the weights given by the coherence-theoretic algorithm^12,13^.

It is also possible to measure the weights from a collimated beam in the far-zone of the waveguide output. The k-space (far-field) expression of the modes is simply given by the Fourier transform – which we denote with a tilde – as in

5 $$\begin{aligned} \tilde{\phi }_p(k_x) = \frac{1}{2\pi }\int _{-\infty }^\infty \phi _p(x)\exp \left( -ik_xx\right) \textrm{d}x. \end{aligned}$$

Similarly, the k-space representation of the total field exiting the waveguide is

6 $$\begin{aligned} \tilde{E}_\textrm{out}(k_x) = \frac{1}{2\pi }\int _{-\infty }^\infty E_\textrm{out}\exp \left( -ik_xx\right) \textrm{d}x. \end{aligned}$$

Since Fourier transform is a linear operation, we immediately see that

7 $$\begin{aligned} \tilde{E}_\textrm{out}(k_x)=\sum _{p=0}^{P-1} \alpha _p\tilde{\phi }_p(k_x), \end{aligned}$$

and the orthogonality of the modes is preserved, so that we may write with the use of (1)

8 $$\begin{aligned} \alpha _p = \int _{-\infty }^\infty \tilde{E}_\textrm{out}(k_x)\tilde{\phi }_p(k_x)\textrm{d}k_x. \end{aligned}$$

Hence, the same weights are obtained by near- and far-field measurements, as one would expect. The present formulation can trivially be extended to 2D modes.

The technique of finding the unknown modal weights of the output field has a remarkable formal similarity to the overlap integral method for input coupling of coherent fields. This method allows one to calculate the weights $\alpha _p$ in end-coupling of a known fully coherent incident field $E_\textrm{in}(x)$ into the waveguide, as in

9 $$\begin{aligned} \alpha _p = \int _{-\infty }^\infty E_\textrm{in}(x)\phi _p(x)\textrm{d}x. \end{aligned}$$

The weights given by (9) are the projections of the incident field onto the waveguide-mode basis. These coefficients depend on polarization (TE or TM) of the incident field since the waveguide modes do so. Now the incident field is expanded in the modal basis of the waveguide as

10 $$\begin{aligned} E_\textrm{in}(x)=\sum _{p=0}^{P-1} \alpha _p\phi _p(x) + E_\textrm{r}(x), \end{aligned}$$

where $E_r(x)$ contains the uncoupled radiation modes. That is, depending on the input field, a varying fraction of the energy in the incident field is lost in the coupling process. If we denote the coupled field as $E_\textrm{c}(x)$, then we can write

11 $$\begin{aligned} E_\textrm{c}(x) = E_\textrm{in}(x) - E_\textrm{r}(x). \end{aligned}$$

The coupled field then propagates through the waveguide to produce $E_\textrm{out}(x)$. However, there is no straightforward general relation between $E_\textrm{c}(x)$ and $E_\textrm{out}(x)$. Although the modes themselves propagate undisturbed by definition, they can lose or gain energy from other modes, depending on the waveguide geometry, refractive index variations, strain, or other imperfections.

The term overlap integral method arises because (9) can be interpreted as a measure of the overlap between the incident field and a given waveguide mode. If we have perfectly matched incident field and some mode *p*, such that $E_\textrm{in}(x) = \phi _p(x)$, we have only one mode for which $\alpha _p=1$ and all others vanish. Additionally, there is no uncoupled radiation and thus the efficiency of the coupling is unity. It is also obvious that the weights of the excited modes depend on, e.g., defocusing, tilting, or misaligning the incident field. For example, if the amplitude of the incident field matches that of mode *p* but the incident field has phase curvature due to defocusing, then $\alpha _p < 1$ and other propagating waveguide modes (as well as radiation modes) are excited as well.

It should be noted that the overlap integral method is not exact for end-coupling. It ignores reflection from the waveguide end (or end facets) and the evanescent part of the in-coupled field. These aspects have been studied in^27^. However, our analysis is completely based on the total output field, and can be generally applied.

Note that this formulation does not only cover end-butt coupling from free space (focused fields in the waveguide input) but also waveguide-to-waveguide (or fiber-to-fiber) coupling. One just needs to know the incident field at the input plane of the waveguide. However, we are interested in the relative mode strengths of the total output field. In other words, how much energy is in a particular mode relative to other modes. In order to find the answer, in practice, one needs to measure the total (complex) electric field distribution $E_\textrm{out}$ at the exit plane. Usually, the phase measurement of total electric field (both the amplitude and phase) requires a reference beam, where the phase profile can be estimated from the interference pattern, e.g., the Twyman-Green interferometer^28^. These types of phase measurement schemes are not trivial while considering the total electric field at the output of a waveguide. Here we present an alternative scheme that utilises a mirror based WFI ^25,26^ to measure the complex valued two-dimensional cross-spectral density function, from which both the amplitude and phase profiles of the total field at the exit plane of a slab waveguide can be calculated. Notably, this method is self-referencing.

## Complex field and modal strength retrieval

The cross spectral density (CSD) for a general scalar field *E*(*x*) is defined as

12 $$\begin{aligned} W(x_1,x_2) = \langle E^*(x_1) E(x_2) \rangle , \end{aligned}$$

where $E(x_i)$ denote the electric field at coordinate $x_i$, the angle brackets denote ensemble averaging, and asterisk denotes the complex conjugate. The CSD is widely used while dealing with partially coherent fields in the space-frequency domain, see e.g., Ref. ^29^. Physically, $W(x_1,x_2)$ represents the correlation between the electric fields at coordinates $x_1$ and $x_2$, at a given frequency. However, for a completely coherent field, such as $E_\textrm{out}(x)$, the CSD can be written in the factored form^30,31^

13 $$\begin{aligned} W(x_1,x_2) = E_\textrm{out}^*(x_1) E_\textrm{out}(x_2), \end{aligned}$$

where the ensemble average vanishes. This is because all realizations of $E_\textrm{out}(x)$ are identical, in which case averaging over all realizations is the same as having only one realization. In terms of the amplitudes $E_0(x)$ and phases $\varphi (x)$, this can be written as

14 $$\begin{aligned} W(x_1,x_2) = E_0(x_1) E_0(x_2) \exp \left\{ -i \left[ \varphi (x_2)-\varphi (x_1)\right] \right\} , \end{aligned}$$

**Fig. 2:**
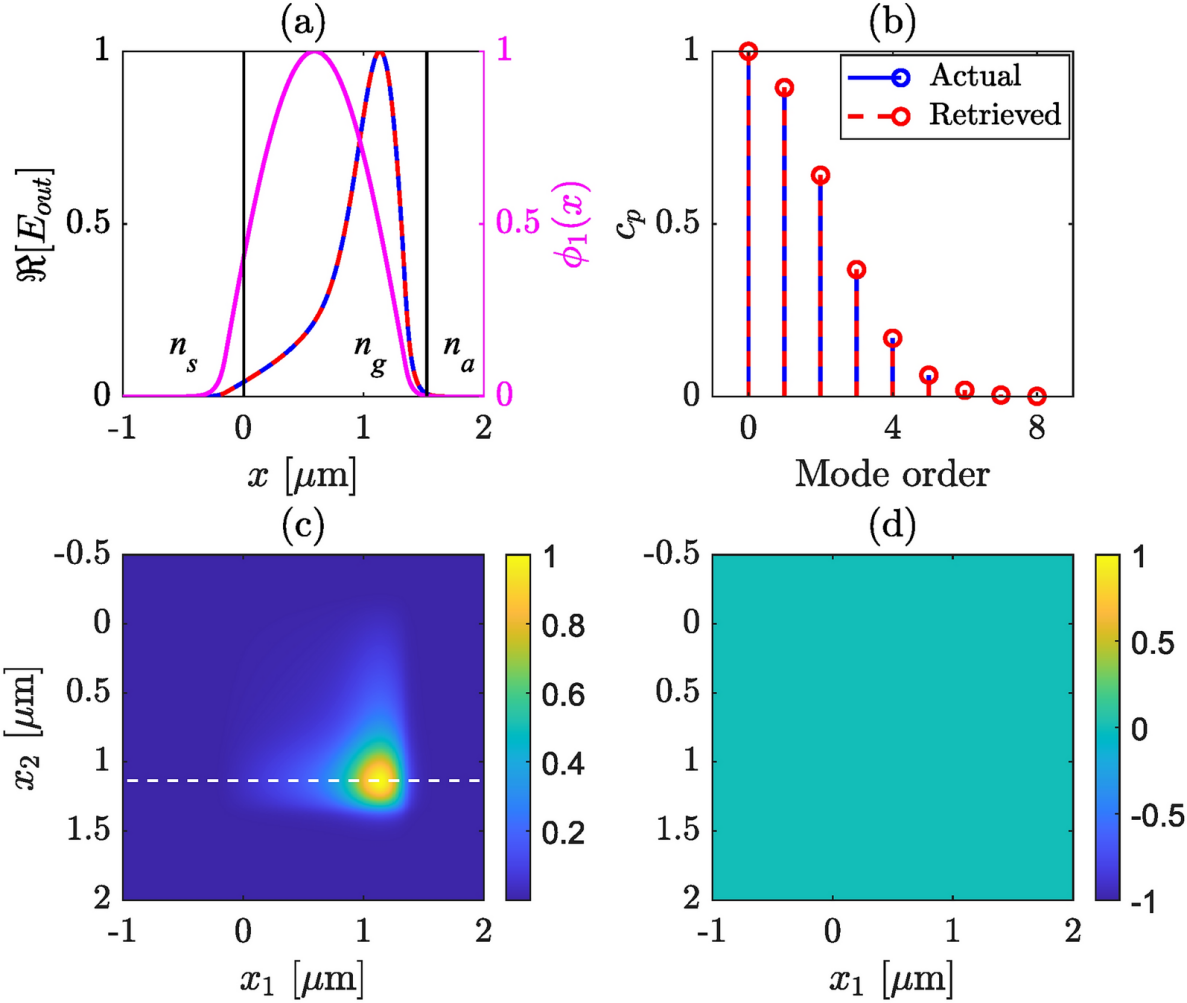
Retrieved modal strengths and output field profile for an asymmetric slab waveguide, for which we consider only the TE modes. The lowest order mode has maximum strength. In (**a**), magenta line shows the fundamental mode, black lines delimit the waveguide boundaries, red and blue lines show real part of the actual and retrieved total field profiles, respectively. (**b**) actual (blue) and retrieved (red) mode strengths are compared, (**c**) real part of CSD, (**d**) phase of CSD. The white dash line in (**c**) indicate the selected line profile of CSD used for mode strength retrieval.

**Fig. 3:**
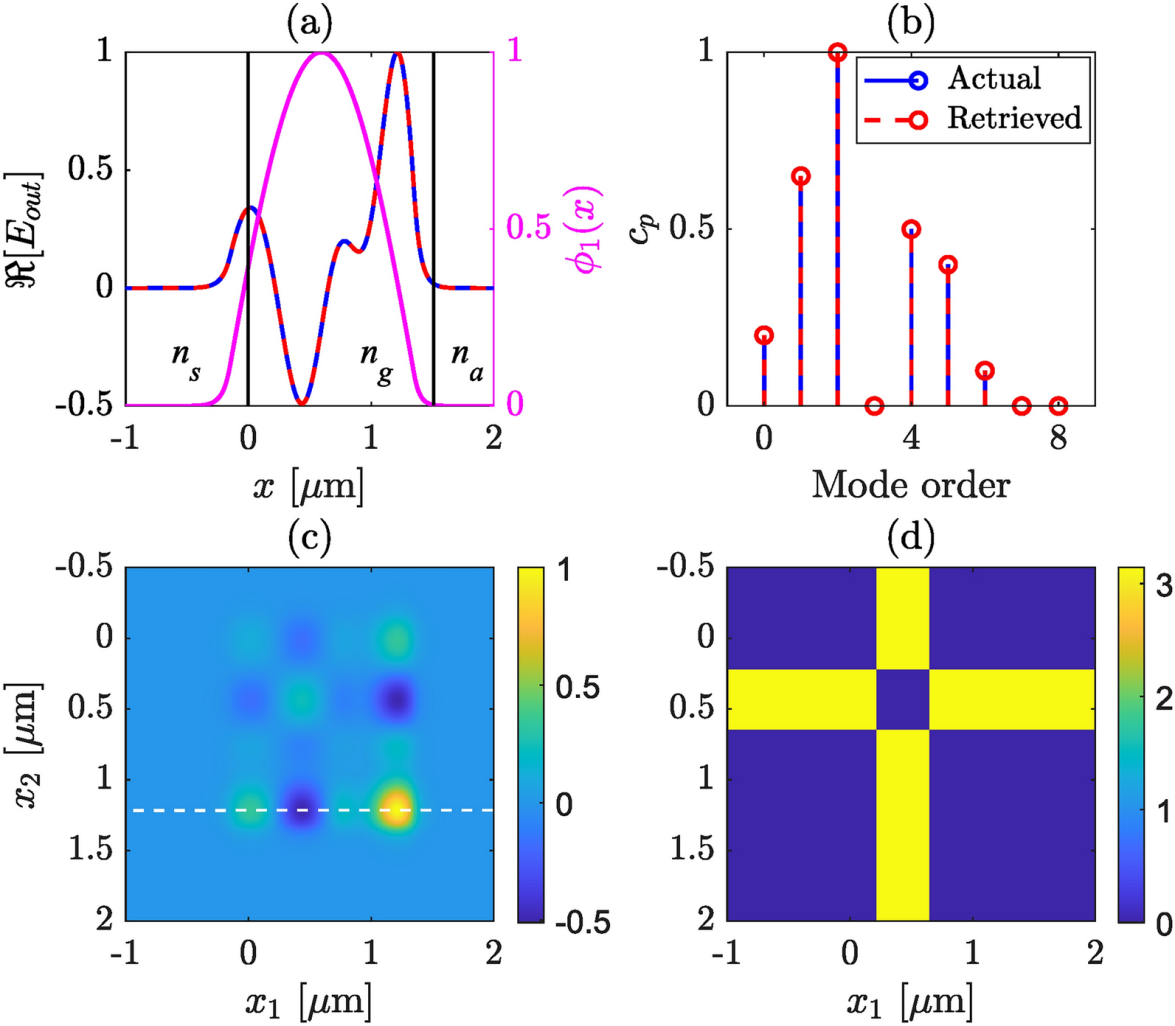
Retrieved modal strengths and output field profile for an asymmetric slab waveguide, for which we consider only the TE modes. The third order mode has maximum strength. In (**a**), magenta line shows the fundamental mode, black lines mark the waveguide boundaries, red and blue lines show real part of the actual and retrieved total field profiles, respectively. (**b**) actual (blue) and retrieved (red) mode strengths are compared, (**c**) real part of CSD, (**d**) phase of CSD. The white dash line in (**c**) indicate the selected line profile of CSD used for mode strength retrieval.

One can then retrieve the phase information of the total field $E_\textrm{out}$ from the CSD, and the amplitude can be calculated from the measured intensity distribution or from the diagonal elements of $W(x_1,x_2)$. Once the complex field has been retrieved, the modal strengths can be retrieved using (3) and (4). To demonstrate the retrieval method numerically, we present two simulated examples in Figs. 2 and 3.

In addition, we note that in the case of two-dimensional (2D) field *E*(*x*, *y*) the CSD is a four-dimensional (4D) function $W(x_1,y_1,x_2,y_2)$ and can be measured using WFI, see Ref. ^26^. Therefore, our method can be generalized for such fields, that has both *x* and *y* dependency.

In both cases we start by defining the slab waveguide parameters, i.e., the thickness of the guiding layer is $h=1.5~\upmu$m and the refractive indices of the cladding, guiding layer, and substrate materials are $n_a = 1$, $n_g = 2.04$, and $n_s = 1.46$, respectively, while the wavelength of the illumination is 520 nm. We calculate the possible/allowed modes and their profiles by solving Maxwell’s equations within the waveguide structure. For an asymmetric planar waveguide with the above parameters, the modes $\phi _p$ are of the form ^32,33^

15 $$\begin{aligned} \phi _{y,p} = {\left\{ \begin{array}{ll} A_p e^{(\rho _p x)}, & \text {for } x \le 0, \\ A_p \cos (\kappa _p x) + B_p \sin (\kappa _p x), & \text {for } 0 \le x \le h, \\ \left[ A_p \cos (\kappa _p h) + B_p \sin (\kappa _p h) \right] e^{-\sigma _p (x - h)}, & \text {for } x \ge h, \end{array}\right. } \end{aligned}$$

while the propagation constants of the modes can be found from the following transcendental equation

16 $$\begin{aligned} \tan (\kappa _p h) = \frac{\kappa _p (\sigma _p + \rho _p)}{\kappa _p^2 - \sigma _p \rho _p}, \end{aligned}$$

where $\sigma _p^2 = \beta _p^2 - k_0^2 n_a^2$, $\kappa _p^2 = k_0^2 n_g^2 - \beta _p^2$, and $\rho _p^2 = \beta _p^2 - k_0^2 n_s^2$. Here, $\phi _{y,p}$ represents the electric field component of the *p*-th mode polarized along the *y*-axis, where $A_p$ and $B_p$ denote the amplitude coefficients of the mode. The variable *x* signifies the spatial coordinate perpendicular to the waveguide layers, with *h* denoting the guiding layer’s thickness. The constants $\rho _p$ and $\sigma _p$ correspond to the decay rates in the substrate and cladding, respectively, while $\kappa _p$ represents the transverse wavenumber in the guiding layer. Lastly, $\beta _p$ is the mode’s propagation constant and $k_0$ the free-space wave number.

Equation (15) represents how the electric field varies along the transverse plane perpendicular to the direction of propagation, chosen here as the *z*-axis. The solutions of Eqs. (15) and (16) yields the the electric field profiles as well as the propagation constants of the waveguide modes. Once the allowed modes are obtained, their properties such as field distribution, effective refractive index, and mode confinement can be analyzed. The confinement factor here refers to the proportion of optical power that is confined within the various regions of the waveguide.

To demonstrate the retrieval method we present two simulated examples in Figs. 2 and 3. The modal strengths, $c_p$, are chosen to follow a Gaussian distribution for Fig. 2, i.e., an ideal coupling in the waveguide to excite mainly the fundamental TE$_0$ mode with all $\alpha _p$ being real valued. The total field (actual) is then calculated following (1), and the real part is shown in panel (a), while the corresponding modal weights are depicted in panel (b) in blue solid line. We calculate the corresponding CSD using (13), the amplitudes and the phases of the CSD are presented in sub figures (c) and (d), respectively. For the Gaussian modal strength distribution the phase is flat, as can be seen from Fig. 2d. Note that, in this particular case, one can construct the total electric field only from the measured intensity across the exit plane of the slab waveguide, since the phase profile is constant. Next, we select a line profile as a function of $x_1$ from the CSD (at a fixed $x_2$) where $|W(x_1,x_2)|$ is maximum, indicated as the white dashed line in (c). This one dimensional (1D) cut of $W(x_1,x_2)$ provides the complex valued total electric field. Using (3), we then retrieve all the $\alpha _p$ values and the modal strengths, $c_p$. The retrieved total field and modal strengths are plotted along with the actual ones for comparison in (a) and (b), respectively, in red dashed lines. In all of the illustrations, the 0th order mode is the same as the fundamental mode.

We follow the same steps for simulating Fig. 3 except we choose random modal strengths. In practice, the random modal strength distribution may arise from several reasons, e.g., defects of the waveguide structure, tilt or miss-aligning the in-coupling field, etc. In this situation the CSD is complex valued and the phase plays a crucial role. Moreover, since we choose all the $\alpha _p$ values real, the phase of CSD and the total field have a binary nature, which is evident from Eqs. (1) and (14) since all $\phi _{p}(x)$ values have a binary profile, see also Fig. 3d. In both illustrations the actual and retrieved modal strengths match and so does the electric field profile. This validates our algorithm for the modal strength retrieval from simulated field distributions. The complex valued $W(x_1,x_2)$ can be measured in practice using a WFI or a digital micro-mirror device (DMD) ^34^. We note that in both cases we select a 1D cut (as marked by white dashed line in Figs. 2c and 3c) of CSD where $|W(x_1,x_2)|$ is maximum. This particular choice is important from the experimental point of view, which will become clear in Sect. 5, where we show the detailed experimental procedure and results.

## Experimental results

**Fig. 4:**
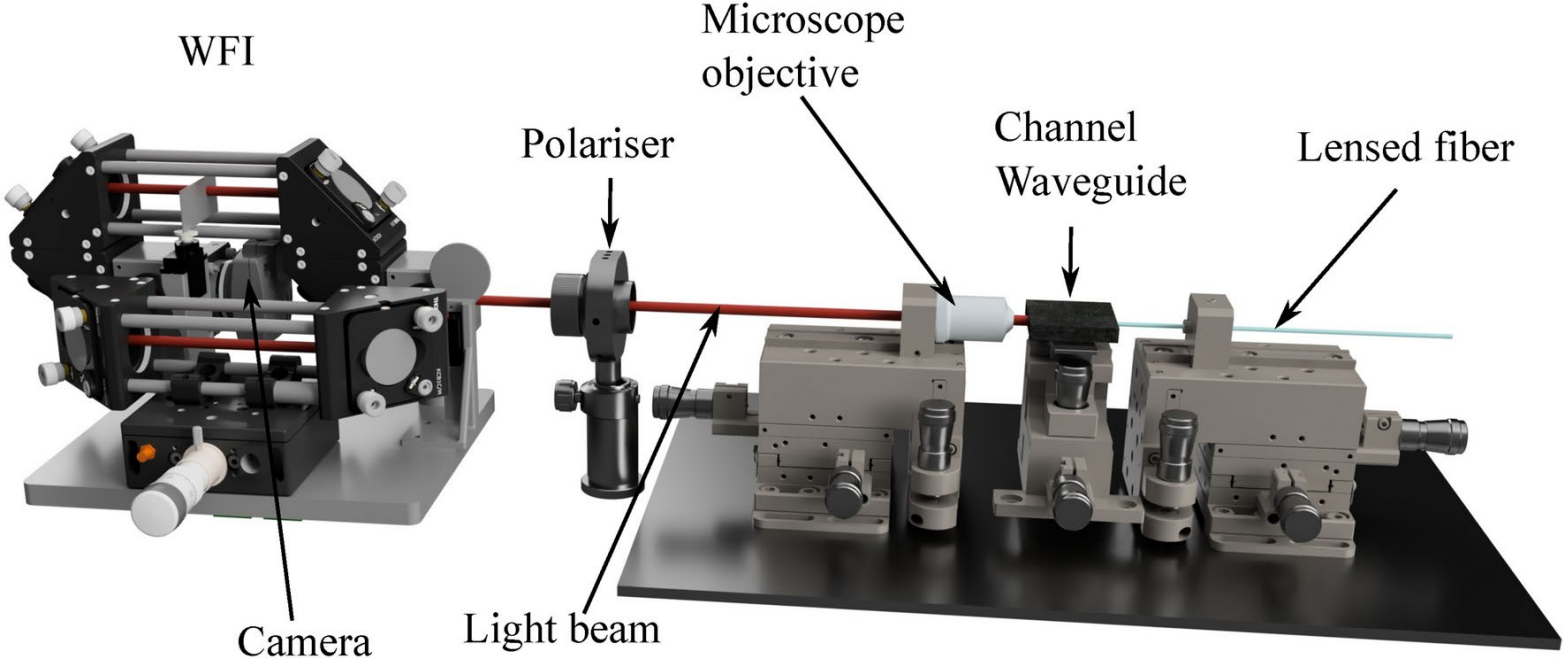
Schematic of the experimental setup showing the WFI on the left, the polariser and the waveguide stage. The lensed fiber is connected to a fiber-coupled laser source from ThorLabs, which is not shown here.

In the following experiments, we demonstrate the feasibility of retrieving specific modes of a silicon nitride channel waveguide illustrated in Fig. 1. This waveguide, measuring 3000 nm in width and 330 nm in height, is fabricated on an oxidized silicon wafer. The wafers with LPCVD silicon nitride layer are specially customized and ordered from Silicon Materials (Germany). The waveguides are fabricated by electron-beam lithography of the nLOF resist (AZ2070, Microchemicals, Germany) and followed by reactive ion etching of the silicon nitride layer through the patterned resist. Effective index calculations for such waveguide using Finite Difference Eigenmode (FDE) solver in Ansys Optics indicate the existence of over 20 modes each for transverse electric (TE) and transverse magnetic (TM) polarizations at $\lambda = 520$ nm. However, it is important to note that not all these modes are efficiently guided through the waveguide due to potential losses. In the following, we consider only the TE polarization state selected with a linear polarizer at the output of the waveguide.

**Fig. 5:**
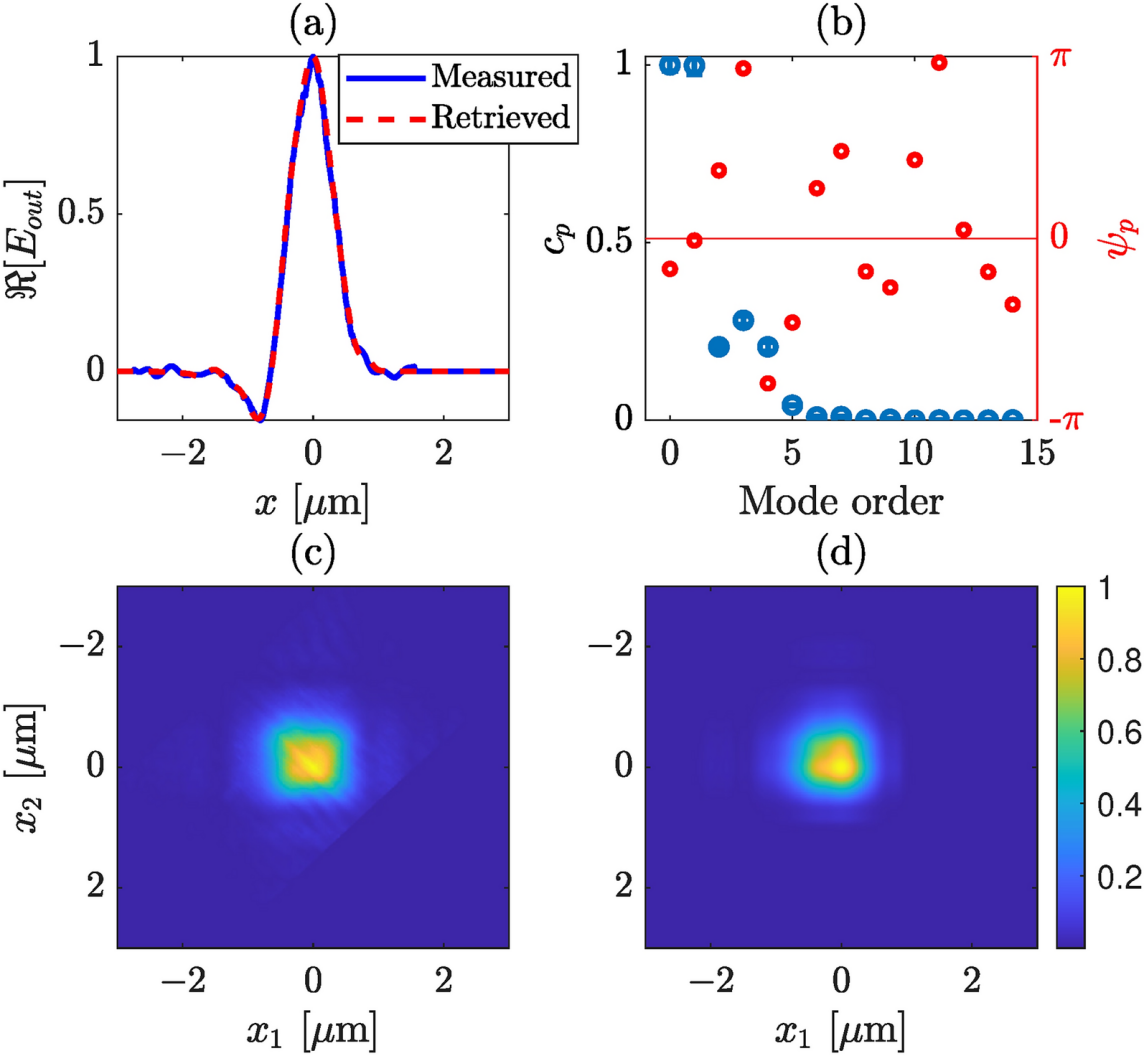
Retrieved modal strengths and output field profile from a silicon nitride waveguide, where we consider only TE modes. (**a**) Measured (blue) and retrieved (red) intensity profiles of the total field at the output plane of the waveguide, (**b**) retrieved mode strengths (blue) and phases (red), (**c**) measured, and (**d**) retrieved absolute CSD. The first two modes have the highest contribution. The error bars correspond to one standard deviation.

**Fig. 6:**
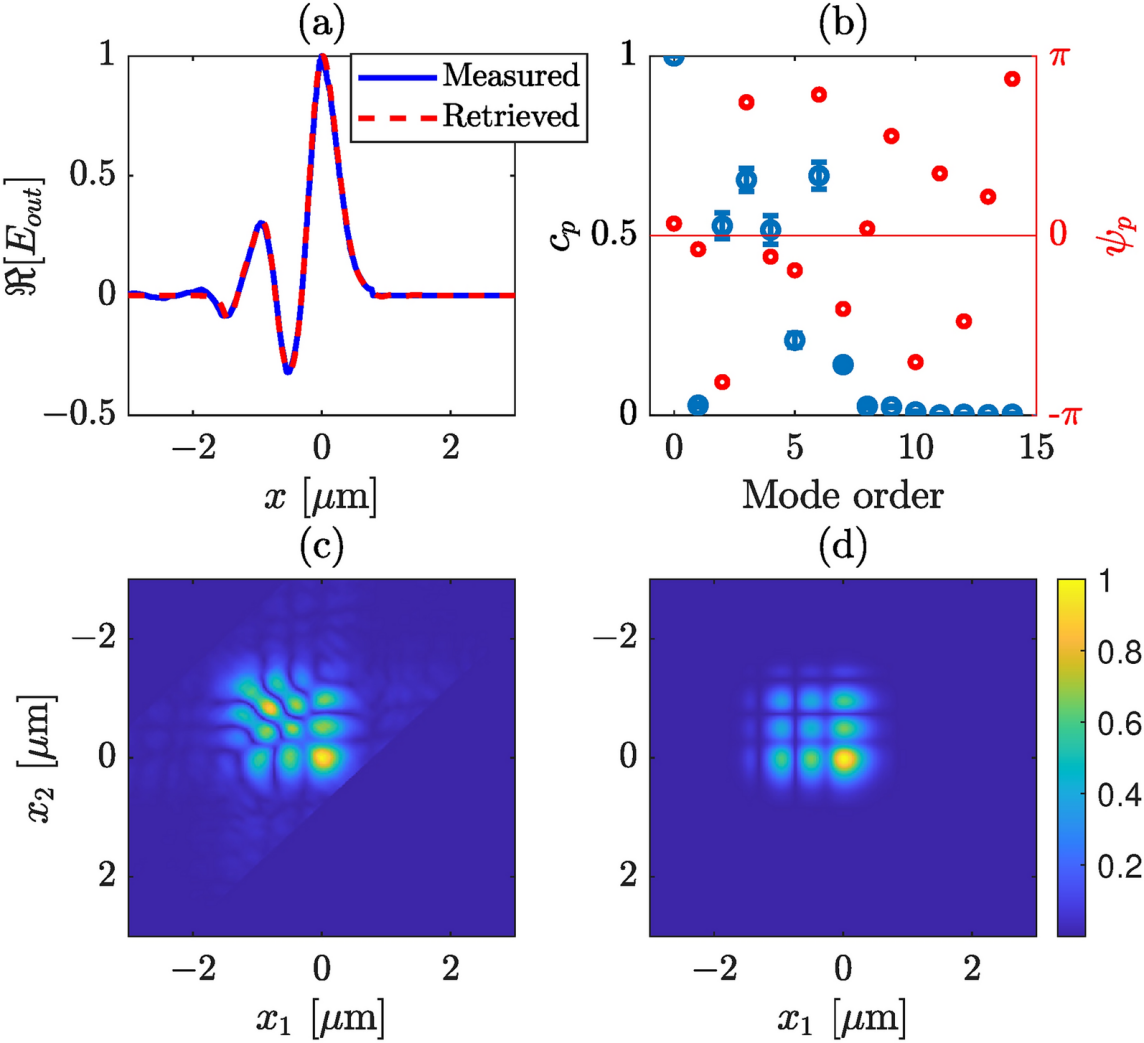
Retrieved modal strengths and output field profile from a silicon nitride waveguide, where we consider only TE modes. (**a**) Measured (blue) and retrieved (red) intensity profiles of the total field at the output plane of the waveguide, (**b**) retrieved mode strengths (blue) and phases (red), (**c**) measured, and (**d**) retrieved absolute CSD. The zeroth mode has the largest contribution, while the 2nd, 3rd,4th, and 6th have weights higher than 0.5. The error bars correspond to one standard deviation.

A schematic of the experimental setup for the mode measurement is shown in Fig. 4. We use a fiber coupled laser source (MCLS1 controller together with a custom laser diode from ThorLabs). We inject light from a finite bandwidth (1.2 nm) continuous wave laser having a central wavelength of 520 nm into the channel waveguide using a tapered lens fiber (focused spot size of $2\pm 0.5~\upmu$m). This butt-coupling involves aligning the input end facet of the waveguide with the optical fiber in such a way that light can be transferred with minimal loss, despite a modal mismatch between the fiber and the channel waveguide. The butt-coupling technique also allows us to laterally shift the fiber or tilt it with high precision, thus altering/adjusting the modal strength distribution, i.e., exciting preferentially the fundamental or the higher order modes. Once the in-coupling is done, the output plane of the channel waveguide is imaged (with sufficient magnification) onto the WFI camera. We use a microscope objective (Olympus Plan N, $\textrm{NA} = 0.65$, 40$\times$) for the imaging purpose. We place a high quality linear polarizer (Glan-Thompson GTH10M polarizer from Thorlabs) to allow only the TE modes.

Next, a WFI is used to capture an interference pattern while keeping all the other components in the setup fixed. The WFI measures the complex valued CSD in average and difference coordinate: $(\bar{x}, \Delta {x})$, which is then mapped to absolute coordinates $(x_1, x_2)$. See, Refs. ^25,26^ for details on the measurement. Any 1D cut from the measured $W(x_1, x_2)$ for a fixed value of $x_2$ (or $x_1$) can be used for retrieving the modal strength distribution and the modal dispersion. However, we choose the 1D cut such that it passes through a point where $|W(x_1, x_2)|$ is maximum to maximize the signal-to-noise ratio. The field retrieved in this way represents the output field with at most a uniform pi phase shift ambiguity. However, this does not affect mode retrieval.

Our experimental setup, as illustrated in Fig. 4, reveals some limitations in the mode detection. Specifically, the number of observed and measured modes is constrained by the NA of the microscope objective used to image the waveguide’s output plane onto the WFI. The finite numerical aperture of the microscope objective (and overall imaging system) limits the number of modes to be collected. This comes from the simple fact that the divergence of each mode increases with the mode order. Modes with propagation angles exceeding the microscope objective’s convergence angle are truncated, making the capture of all propagating modes highly challenging, if not impossible. More developed imaging system can be used in order to increase the collection angle. However, this is not required to validate our method.

**Fig. 7:**
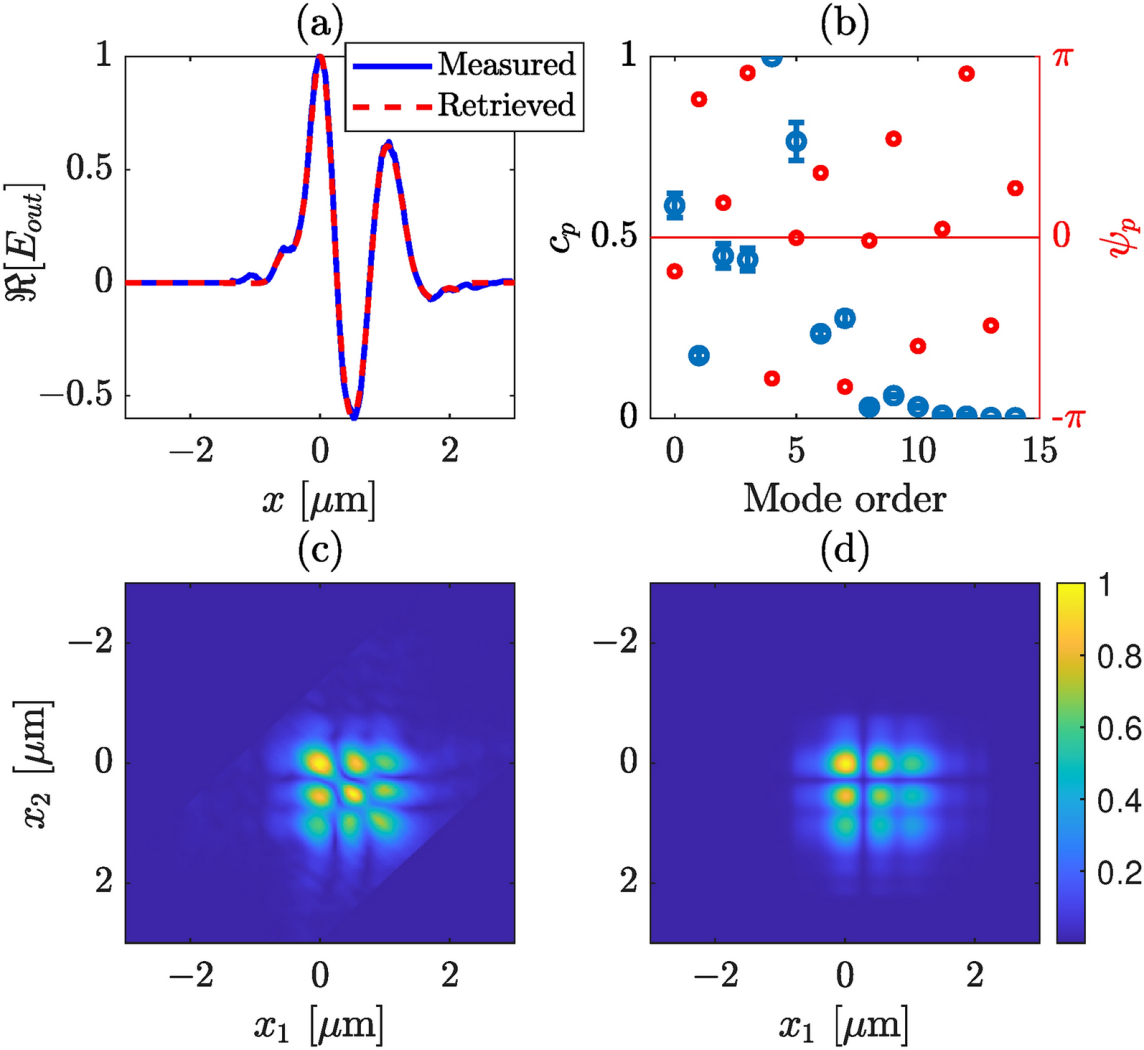
Retrieved modal strengths and output field profile from a silicon nitride waveguide, where we consider only TE modes. (**a**) Measured (blue) and retrieved (red) intensity profiles of the total field at the output plane of the waveguide, (**b**) retrieved mode strengths (blue) and phases (red), (**c**) measured, and (**d**) retrieved absolute CSD. The 4th order mode has the largest contribution, while the fundamental, 2nd, 3rd and 5th order modes have weights around or over 0.5. The error bars correspond to one standard deviation.

An interesting aspect of our experiment is the ability to adjust the mode order with the highest weight simply by shifting the lensed fiber laterally or by altering the injection angle. This versatility is demonstrated in Figs. 5, 6, and 7, which represent different excitation conditions for TE modes. For each case, the mode strengths have been measured and quantified in the same $\textrm{Si}_3\textrm{N}_4$ waveguide as previously discussed. It is important to highlight that although more than 20 modes were used for calculating mode strengths, only the first 15 modes are presented, as the contributions of higher-order modes are either zero or negligible.

In Fig. 5a both the measured (in blue) and the retrieved (in red) electric field profile (real part) are presented. The retrieved complex valued electric field is constructed using the normalized strength of modes as shown in Fig. 5b, where the normalized modal weight (in blue circles) and associated phases (in red circles) of each of the first 15 quasi-TE modes is illustrated. The agreement between the measured and retrieved field profiles indicates the robustness of the algorithm. Figure 5c showcases the measured CSD, while Fig. 5d displays the retrieved CSD at the output plane of the channel waveguide utilizing the retrieved mode strengths.

A consistent display arrangement is maintained for various cases, as demonstrated in Figs. 6 and 7 for the same channel waveguide with different injection condition. A distinction can be made between Figs. 5c, 6c, and 7c, which is due to the variations in the modal strength of individual modes during measurements. The modal strength distribution is altered by shifting the lens fiber along the transverse plane slightly. In Fig. 5b, the fundamental mode exhibits the highest strength, followed by the first-order mode. Contributions from other modes (3rd–5th) are minimal, while others are approaching zero. Similarly, in Fig. 6, the fundamental mode dominates in strength, although the first-order mode vanishes this time. Several other higher-order modes have weights greater than, or equal to 0.5. The strength of higher-order modes in Fig. 6b is significant and adds more features in the total electric field, unlike the scenario in Fig. 5b.

**Fig. 8:**
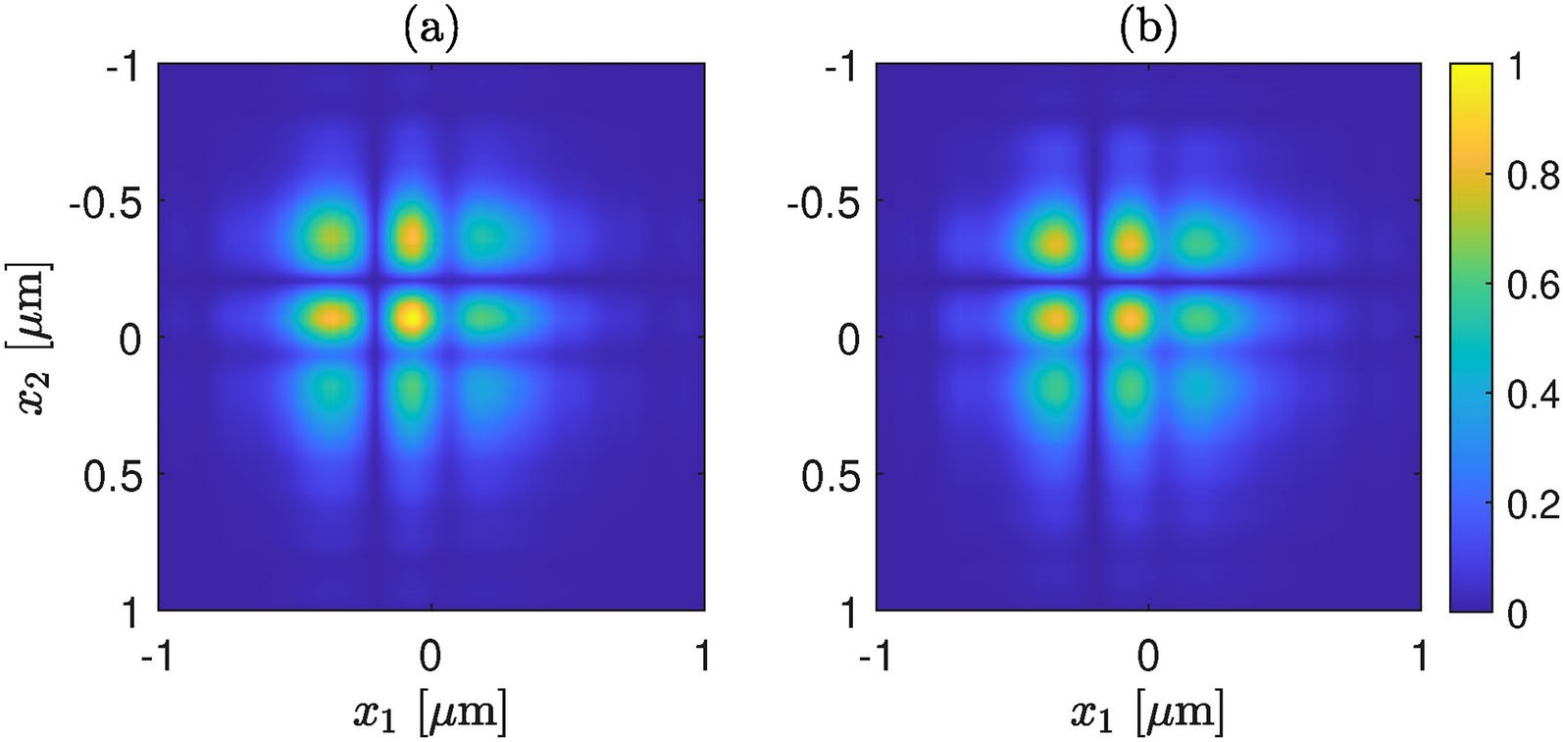
Illustration of the effect of propagation CSD, (**a**) at $z = 0$, and (**b**) at $z = 2~\upmu$m.

In Fig. 7b, the fourth-order mode is dominating followed by the 5th order, whereas the fundamental, 2nd, and 3rd-order modes have similar strengths. Notably, the contribution of higher-order modes is substantial in the last two illustrations. In all the cases, we find good agreement between the retrieved and the measured electric field profiles, as illustrated in Figs. 5a, 6a, and 7a. However, a slight disagreement can be observed between the retrieved and measured CSDs. We note that although in the retrieval algorithm we have considered a strictly monochromatic field, in practice the laser we used has (Lorentzian shaped) finite bandwidth, a full width at half maximum of 1.2 nm. In addition, although in our analysis we have considered that a perfect image of the exit plane of the waveguide is captured by the WFI there might be a couple of micron error in the object plane. In practice, this error could arise from the depth of focus of the microscope objective or/and from the manual adjustment of mechanical stage that holds the objective. This mean that the measured CSD is not perfect, rather a slightly propagated CSD. This effect is illustrated in Fig. 8, where in (a) we show the CSD at the exit plane (at $z = 0$), and in (b) at $z = 2~\upmu$m. A slight stretching in the propagated CSD can be observed, which we believe also describes the discrepancies between the retrieved and measured CSDs in Figs. 5, 6, and 7.

In all of the presented cases, we calculate the error bars associated with the modal strengths by numerically adding random number distribution - with the range of $\pm 12.5\%$ of peak amplitude value - with the amplitude bins of the measured electric field. Note, however, that our measured CSDs do not feature such large noise and the error bars computed in this way represent a worst case scenario.

The error bars represent one standard deviation from the mean, calculated from 20 independent realizations of numerically calculated electric field $E_\mathrm{{out}}$, in each case. In practice, these may arise from low signal conditions. The error bars show the robustness of the presented scheme. Finally, from the retrieved phase distribution associated with each modal strength (since $\alpha _p$ values are complex), the dispersion between the different modes can be estimated. This dispersion is due to both the in-coupling condition and different propagation constants of different mode orders.

## Conclusion

In summary, this paper introduces an innovative method for determining mode strengths in arbitrary asymmetric channel waveguides by measuring the complex electric field distribution at the exit plane using WFI. The proposed technique, demonstrated experimentally with a silicon nitride channel waveguide, provides a robust approach for estimating modal strengths in spatially coherent light, where guided modes exhibit correlation, resulting in spatially completely coherent output. Our approach showcases adaptability, allowing for the adjustment of mode order with the highest intensity by manipulating the injection angle. However, acknowledging limitations, such as mode truncation due to microscope objective constraints, opens avenues for future experimental setups to comprehensively observe all propagating modes. Furthermore, these investigations can be extended to the incoherent case, broadening the applicability of the proposed method and providing deeper insights into waveguide characteristics. Our study contributes to the understanding and characterization of guided modes in channel waveguide design. The presented results and methodology offer valuable insights for researchers and engineers in the field of integrated optics and photonics, showcasing promise for optimizing device performance in applications such as optical communication and sensing.

Finally, we also emphasize that the mode strength retrieval based on measured intensity distribution is not unique. However, here we demonstrate an alternative approach where the retrieval is based on the measured complex valued CSD. Since we can get complete information on both the amplitude and phase profile of the electric field from the complex-valued CSD, our retrieval relies on the measured complex-valued electric field. Therefore, this method provides a unique solution. Moreover, the inter-modal dispersion properties of a waveguide can also be estimated via our measurement scheme. The presented error analysis also shows the robustness of our technique. Such a technique can easily be implemented in any integrated optics characterization system, even at the industrial level.

## Data Availability

The datasets generated during and/or analysed during the current study are available from the corresponding author on reasonable request.
